# Minimal conformational plasticity enables TCR cross-reactivity to different MHC class II heterodimers

**DOI:** 10.1038/srep00629

**Published:** 2012-09-04

**Authors:** Christopher J. Holland, Pierre J. Rizkallah, Sabrina Vollers, J. Mauricio Calvo-Calle, Florian Madura, Anna Fuller, Andrew K. Sewell, Lawrence J. Stern, Andrew Godkin, David K. Cole

**Affiliations:** 1 Institute of Infection and Immunity, Cardiff University School of Medicine, The Henry Wellcome Building, Cardiff, CF14 4XN, United Kingdom; 2 Department of Pathology, University of Massachusetts Medical School, Worcester, MA 01655; 3 Department of Biochemistry & Molecular Pharmacology, University of Massachusetts Medical School, Worcester, MA 01655; 4 Department of Integrated Medicine, University Hospital of Wales, Cardiff, CF14 4XW, United Kingdom; 5 These authors contributed equally.

## Abstract

Successful immunity requires that a limited pool of αβ T-cell receptors (TCRs) provide cover for a vast number of potential foreign peptide antigens presented by ‘self’ major histocompatibility complex (pMHC) molecules. Structures of unligated and ligated MHC class-I-restricted TCRs with different ligands, supplemented with biophysical analyses, have revealed a number of important mechanisms that govern TCR mediated antigen recognition. HA1.7 TCR binding to the influenza hemagglutinin antigen (HA_306–318_) presented by HLA-DR1 or HLA-DR4 represents an ideal system for interrogating pMHC-II antigen recognition. Accordingly, we solved the structure of the unligated HA1.7 TCR and compared it to both complex structures. Despite a relatively rigid binding mode, HA1.7 T-cells could tolerate mutations in key contact residues within the peptide epitope. Thermodynamic analysis revealed that limited plasticity and extreme favorable entropy underpinned the ability of the HA1.7 T-cell clone to cross-react with HA_306–318_ presented by multiple MHC-II alleles.

αβ T-cell receptor (TCR) binding to peptide-major histocompatibility complex class-II (pMHC-II) orchestrates the adaptive immune response^1^. The specificity of this pivotal receptor/ligand interaction is determined by the antigen-binding variable domains of the TCR. These variable domains include six complementarity determining region (CDR) loops that recognize composite ligands comprised of a peptide-antigen in complex with a ‘self’ MHC. The potential array of peptides that can be generated from combinations of the 20 proteogenic amino acids and that have appropriate anchors to enable binding to a self-MHC molecules dwarfs the <10^8^ unique T-cell clonotypes in the naïve human T-cell pool^2^,^3^. As pathogens can evolve many times faster than their host, a vertebrate T-cell system that failed to respond to all of these possibilities could be rapidly overrun. Theoretical and experimental evidence suggests that effective immunity requires each T-cell, and thus each TCR, to be capable of recognising huge numbers of potential antigenic peptides^3^. The mechanisms by which this level of TCR degeneracy is generated are not well understood.

To date, our understanding of the molecular events that occur upon TCR engagement have come, almost exclusively, from MHC class-I (MHC-I)-restricted TCRs. The structures of several TCR-pMHC-I pairings, in bound and unbound form, have shown that MHC-I antigen recognition can be very dynamic. In a number of cases, large CDR loop movements (>5Å) have been reported during TCR binding^4^. Early thermodynamic analyses of these TCR-pMHC-I interactions showed that binding was generally characterized by unfavorable entropy (i.e. transition from a disordered to an ordered state) which was counteracted by favorable enthalpy (i.e. an exothermic reaction mediated by a net gain in electrostatic interactions)^5^. These analyses suggested that conformational plasticity in the TCR CDR loops was energetically favored and played an important role in T-cell antigen recognition and crossreactivity^6^,^7^,^8^. However, more recent studies have shown that the TCR face can remain more rigid^9^,^10^,^11^,^12^,^13^, and MHC-I restricted TCRs can bind using a range of thermodynamic strategies^5^. Furthermore, although the surface of pMHC-Is normally remain conformationally fixed during binding, large changes in the peptide conformation^13^, or MHC-I surface^14^, have been reported to play an important role in some instances of MHC-I restricted T-cell antigen recognition^14^. It is not clear whether such mechanisms extend to recognition of MHC-II-restricted antigens. Understanding of the molecular events involved in pMHC-II recognition has been hampered by the fact that there are no thermodynamic data for human MHC-II restricted TCRs, and only one human TCR-pMHC-II complex has been determined with an accompanying unligated TCR structure^15^. A direct comparison of the bound/unbound E8 TCR showed that the TCR underwent small conformational alterations of several CDR loops upon pMHC-II ligation^15^. However, the relationship of how these conformational changes to the TCR might have influenced the potential degeneracy of the responding T-cell was not investigated^15^.

To explore this question further, we examined TCR-pMHC-II binding degeneracy using the HA1.7 TCR that recognizes an influenza hemagglutinin derived epitope (HA_306–318_). This influenza-derived peptide has been shown to bind to HLA-DRα*0101 in complex with either; HLA-DRβ*0101 (DR1), DRβ*1501, DRβ*0401 (DR4), DRβ*0404, DRβ*0501 or DRβ*0701 and is, therefore, a so called ‘universal’ antigen^16^,^17^,^18^. Curiously, the HA1.7 CD4^+^ T-cell clone recognized HA_306–318 _presented by both DR1 (DR1-HA) and DR4 (DR4-HA)^19^. The differences in amino acid sequence between DR1 and DR4 are located deep in the peptide binding groove, and do not directly contact the TCR. However, other DR1 restricted HA_305–320 _specific TCRs were unable to recognize HA_305–320 _in the context of DR4^20^. Therefore, allelic variations in the peptide-binding groove can influence the immunogenicity of the pMHC-II surface. Additionally, previous investigations have shown that the HA1.7 T-cell clone can recognize multiple peptide ligands in the context of both DR1 and DR4^21^,^22^. Taken together, these data indicate that the HA1.7 T-cell clone can promiscuously bind to different MHC-IIs presenting a wide range of distinct peptide epitopes.

Here, we determined the structure of the unligated HA1.7 TCR in order to understand the molecular rules that allowed it to bind to both DR1 and DR4. We proceeded to perform an analysis of HA1.7 CD4^+^ T-cell activation against a series of peptides with mutations to key contact residues within the HA peptide epitope. Finally a thermodynamic investigation of these interactions demonstrated that the HA1.7 TCR used an extreme energetic strategy during antigen recognition. Overall, these data provide important new information concerning the molecular rules that govern antigen recognition by MHC-II restricted T-cells.

## Results

### Minimal CDR-loop movement enables HA1.7 TCR binding to DR1-HA and DR4-HA

**Figure 1:**
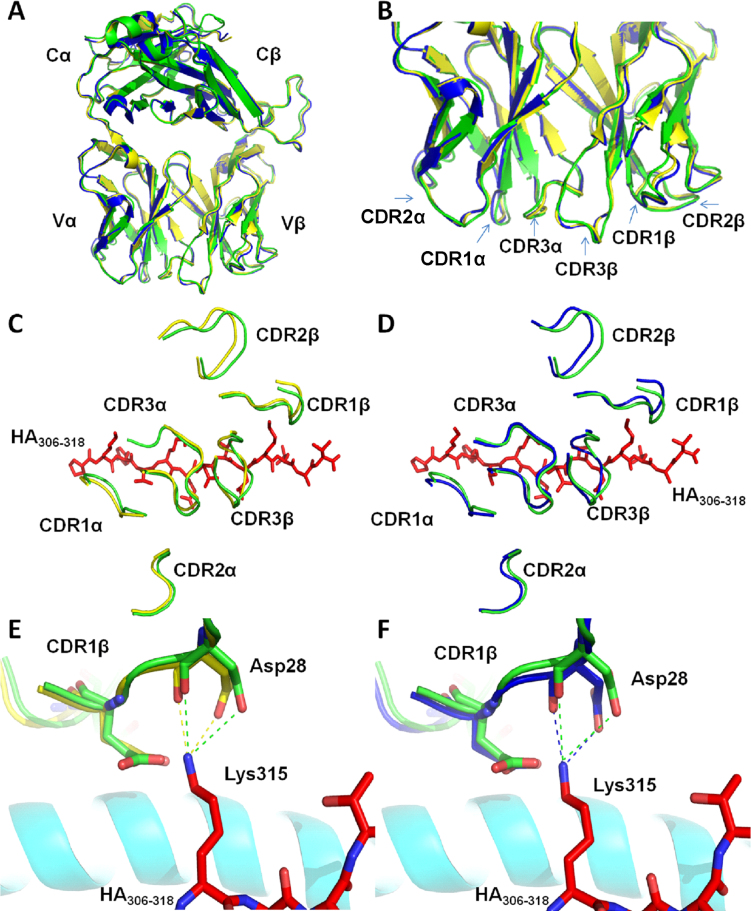
Comparison of the HA1.7 TCR CDR loop conformations pre and post ligation to DR1 and DR4. (A) Superposition of the unligated HA1.7 TCR (green) with the ligated conformations of the HA1.7 TCR when bound to DR1 (yellow) and DR4 (blue). (B) Conformation of the HA1.7 TCR CDR loops comparing ligated and unligated structures (colors as in (A)). (C&D) A view of the superimposed CDR loops from the perspective of the TCR contacting a stick representation of the HA_306–318_ peptide (red). Green loops are the unligated HA1.7 CDR loops, yellow are HA1.7 CDR loops when ligated to DR1 (C) and blue are HA1.7 CDR loops when ligated to DR4 (D). (E&F) HA1.7 CDR1β contacting the HA_306–318 _residue, 315K, presented by either DR1 (E) or DR4 (F). Green loops are the unligated HA1.7 CDR loops, yellow are HA1.7 CDR loops when ligated to DR1 and blue are HA1.7 CDR loops when ligated to DR4. Contact distances were smaller, allowing salt bridge formation, between the TCR and 315K in the TCR ligated forms.

**Figure 2:**
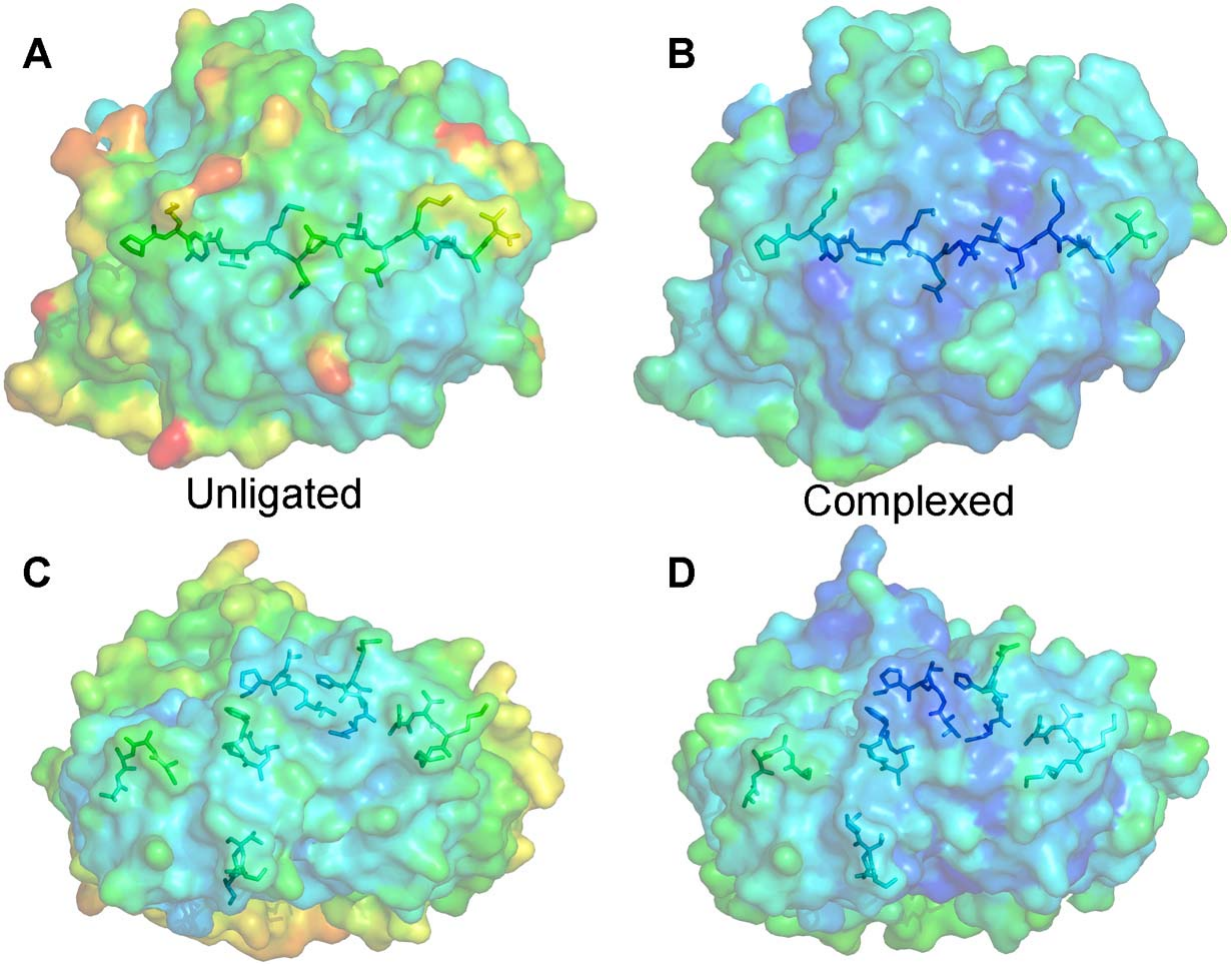
Analysis of free energy comparing the unligated HA1.7 TCR and DR1-HA strutures and the HA1.7-DR1-HA complex. Surface representation of unligated and ligated HA1.7 TCR and DR1-HA colored using B-factors (a PYMOL script that colors residues depending upon their motion) ranging from the greatest degree of motion in red, to least motion in blue. (A) unligated DR1-HA (PDB: 1DLH)^58^, (B) complexed DR1-HA (PDB: 1FYT)^39^, (C) unligated HA1.7 TCR (PDB: 4GKZ) and (D) complexed HA1.7 TCR (PDB: 1FYT)^39^.

To date, only one TCR-pMHC-II complex has been solved in conjunction with the unligated TCR. Theses structures were for an altered-self–reactive TCR of uncertain degeneracy^15^. In order to analyze the degree of conformational plasticity during binding of a degenerate TCR to MHC-II, we determined the structure of the HA1.7 TCR using diffraction data extending to 2.4Å **(**Table 1**)**. The HA1.7 TCR has previously been solved in complex with DR1-HA and DR4-HA and was the first human MHC-II restricted TCR complex to be published^39^,^40^. Thus, this important disease-relevant model system allowed a comparison between ligated and unligated TCR binding in two distinct systems. Molecular replacement was successful in space group P12_1_1, consistent with the presence of one molecule of the complex per asymmetric unit. The resolution was sufficiently high to show the conformation of the HA1.7 TCR CDR loops and contained well-defined electron density throughout the structure. The final model showed 100% of residues in the preferred, or allowed, regions of the Ramachandran plot and geometry consistent with the data resolution. The crystallographic R/Rfree factors were 22.9% and 29.4%, respectively. The ratio was within the accepted limits shown in the theoretically expected distribution^41^. The molecular visualization software, PyMol, was used to perform a secondary structure-based alignment of the unligated HA1.7 TCR with the two solved TCR-pMHC-II complexes, HA1.7-DR1-HA (PDB = 1FYT)^39^ and HA1.7-DR4-HA (PDB = 1J8H)^40^. This allowed direct visualization of the conformation of the CDR loops before and after MHC-II association **(**Figure 1A–D**)**. Measurement of individual CDR loop shifts upon ligation indicated that very little movement (maximum movement = 2.28Å) was required in these regions for association to occur with either DR1-HA or DR4-HA **(**Table 2**)** (calculated as in^4^). The average CDR loop movement observed during association with either DR1-HA or DR4-HA was 1.28Å and 1.52Å, respectively. Comparison of the HA1.7 TCR complex structures demonstrated that the HA1.7 TCR made 4 hydrogen bonds and 8 salt bridges with DR1-HA, compared to 3 hydrogen bonds and 7 salt bridges with DR4-HA **(**Supplementary Tables 1&2**)**. Analysis of the HA1.7 TCR amino acid side chains that formed these key contacts indicated that the CDR loop movements were required to allow formation of ~50% of the HA1.7 TCR hydrogen bonds and salt bridges upon complex formation with either DR1-HA, or DR4-HA **(**Figure 1E&D**)**. Furthermore, a greater degree of movement was required by the HA1.7 TCR to associate with DR4-HA compared to DR1-HA, possibly explaining the difference in binding affinity between the two complexes^42^. The largest difference in conformational movement was observed for the CDR1β loop, moving 2.28Å when in complex with DR4-HA, and only 0.97Å when bound to DR1-HA. This conformational shift allowed 28D of the TCR CDR1β loop to contact HA-315K forming 2 salt bridges in both complexes **(**Figure 1E&F**)**. We then investigated the thermal stability, by studying the B-factor heat plots, for residues involved in the binding interface for the ligated and unligated DR1-HA and HA1.7 molecules (Figure 2). In agreement with our structural analyses, the B-factor heat plots were similar for the ligated and unligated molecules, indicating minimal conformational adjustments during binding. Interestingly, DR1-HA (Figure 2A&B) underwent a greater degree of stabilization, observable by a greater change in the B-factor heat plot, compared to the HA1.7 TCR (Figure 2C&D**)**. Thus, these analyses support the notion that the HA1.7 TCR remains relatively rigid during ligand engagement.

In their previous analyses of the HA1.7-DR1-HA and HA1.7-DR4-HA complexes, Hennecke *et al.* demonstrated that the center and COOH-terminal half of the HA_306–318 _peptide bound deeper in the groove and closer to the β1 α-helix of DR4 compared to DR1^40^. Thus, although only small CDR loop shifts were observed **(**Table 2**),** these movements were essential for binding. Furthermore, it is likely that the plasticity within the HA1.7 TCR CDR loops allowed the TCR to tolerate the conformational differences associated with HA_306–318 _presented by either DR1 or DR4. This specific level of loop plasticity, although small, may be important to allow the HA1.7 TCR to bind to the range of altered peptide ligands (APLs) described herein with adequate strength to enable recognition by the HA1.7 T-cell clone.

### HA1.7 recognizes multiple HA_306–318_ peptide variants

**Figure 3:**
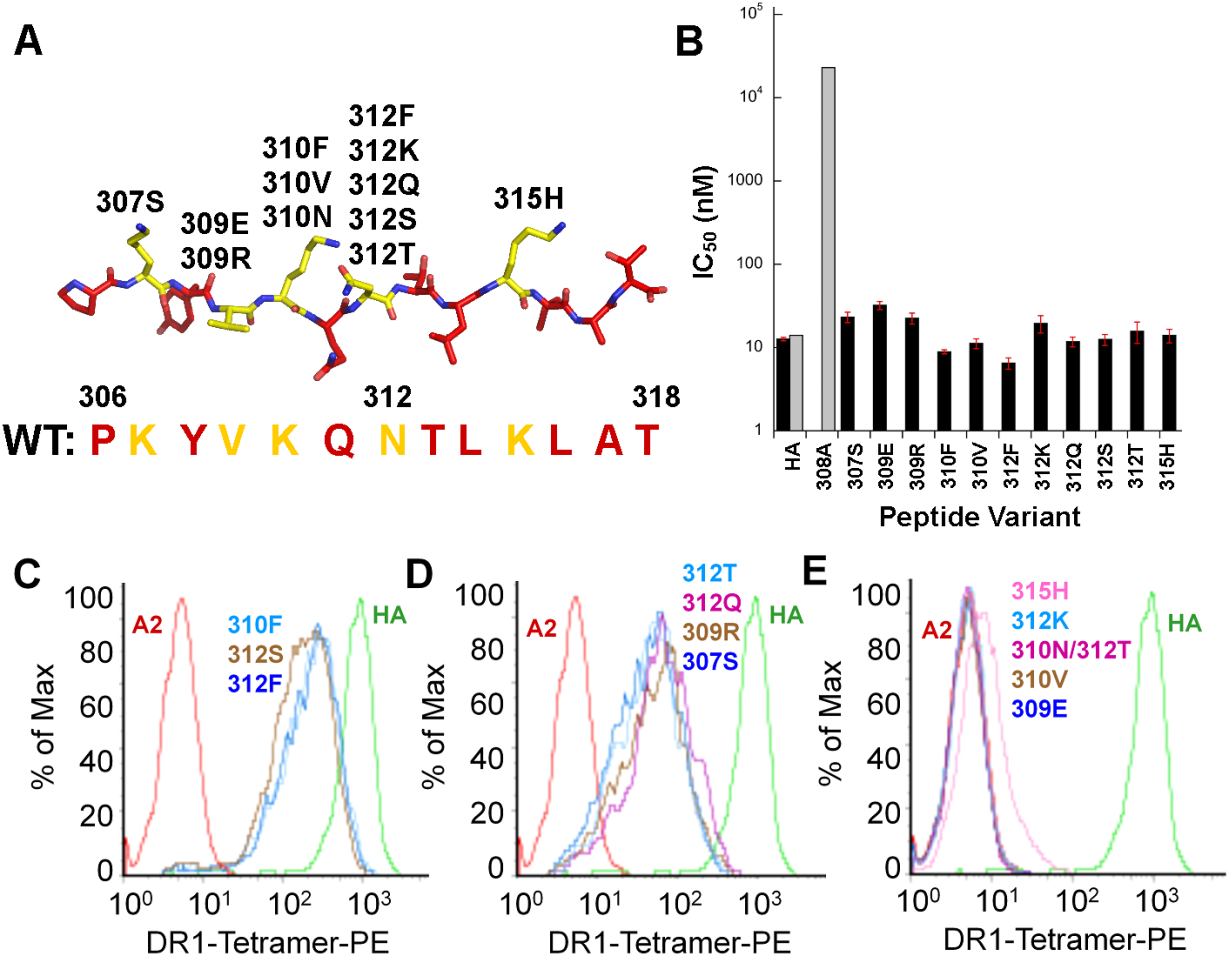
Analysis of altered peptide ligand (APL) stability and tetramer staining of the HA1.7 T-cell clone. (A) Schematic of the presentation of the HA_306–318_ peptide (red and yellow sticks) by DR1. HA1.7 TCR contact residues that were mutated to form the APLs using conservative, semi-conservation and non-conservative mutations are shown as yellow sticks. (B) IC_50_ APL stability assay. The bar in grey shows our previously published observation that the Y308A mutation, that is involved in anchoring the HA peptide into the DR1 binding groove, substantially affects pMHC-II stability^31^. The black bars show our new data that demonstrates that all of the APLs used in this study bind to DR1 with similar affinities, discounting the possibility that peptide stability was the chief factor determining the antigen sensitivity of the HA1.7 T-cell clone. (C–E) HA1.7 T-cell tetramer staining histogram plots using DR1-APL tetramers. (C) Tetramer staining using DR1 bound to a non-cognate A2 peptide, 310F, 312S, 312F and wild-type HA. (D) Tetramer staining using DR1 bound to a non-cognate A2 peptide, 309R, 307S, 312T, 312Q, and wild-type HA. (E) Tetramer staining using DR1 bound to a non-cognate A2 peptide, 315H, 312K, 310N/312T, 310V, 309E and wild-type HA.

Lamb *et al.* previously demonstrated that the hemagglutinin specific CD4^+^ T-cell clone, HA1.7, could cross react with the HA_306–318 _epitope presented by either DR1 or DR4^19^. Furthermore, HA1.7 can recognize APLs in the context of DR1 and DR4^21^,^22^ demonstrating the principle of peptide cross reactivity. Structural investigation by Hennecke *et al.*, into how the HA1.7 clone could achieve this, suggested that the HA1.7 TCR could tolerate the differing conformations of the HA_306–318_ peptide, at positions 310K and 311Q, when presented by DR4 compared to DR1^39^. We therefore set out to determine the degree of functional tolerance of the HA1.7 T-cell clone for previously identified TCR contact residues^39^,^40^. A set of APLs were generated in which key TCR contacts in the HA_306–318_ peptide were mutated (307K, 309V, 310K, 312N & 315K), while key MHC contacts were conserved (Figure 3A**)**^20^,^43^. Importantly, these mutations, that were not at key anchor residue positions, did not substantially influence peptide-MHC stability **(**Figure 3B**).**

We evaluated the ability of the HA1.7 T-cell clone to tolerate substitutions in the peptide ligand at TCR contact residues using a variety of assays. Cell-surface HA1.7 TCR binding of purified pMHC-II complexes was measured using an MHC tetramer staining assay **(**Figure 3C–E**)**. The functional response of HA1.7 T-cells to cell surface MHC-peptide complexes was measured using T-cell proliferation **(**Supplementary Figure 1A**)** and IL-2 secretion **(**Supplementary Figure 1B**)** assays with peptide-pulsed DR1^+^ lymphoblastoid cells used as antigen-presenting stimulator cells (see Methods for details). Each of these assays showed that a range of conservative (312Q), semi-conservative (312S, 312T) and non-conservative (312F), substitutions were tolerated by HA1.7 at asparagine 312 in the centre of the HA_306–318_ epitope, with only substitution for lysine (312K) completely abolishing recognition **(**Figure 3C–E, Supplementary Figure 1**)**. Furthermore, the HA_306–318_ epitope contains three lysine residues at position 307, 310 and 315, which form the majority of hydrogen bonds/salt bridges with the CDR loops of the HA1.7 TCR when bound to either DR1 or DR4, and a valine at position 309 that participates in hydrophobic interactions. Individual substitutions of these dominant contact residues with semi-conservative (307S), and some (309R, 310F) but not all non-conservative (309E, 310V), substitutions were also tolerated by HA1.7 **(**Figure 3C–E, Supplementary Figure 1**)**. Interestingly, a semi-conservative mutation from lysine 315, which forms 4 salt bridges and 1 hydrogen bond with the HA1.7β CDR loops **(**Supplementary Table 1&2**),** to histidine still produced a good receptor agonist **(**Supplementary Figure 1**)**, but the affinity of the interaction was suffciently weak that tetramer staining was barely above background **(**Figure 3E, Supplementary Figure 1**)**. Overall, these data and previous studies^21^,^22^ suggest that HA1.7 T-cells have the ability to recognize with a wide range of different peptide ligands presented by both DR1 and DR4, some with very weak affinities.

### Entropic effects drive HA1.7 TCR antigen recognition

**Figure 4:**
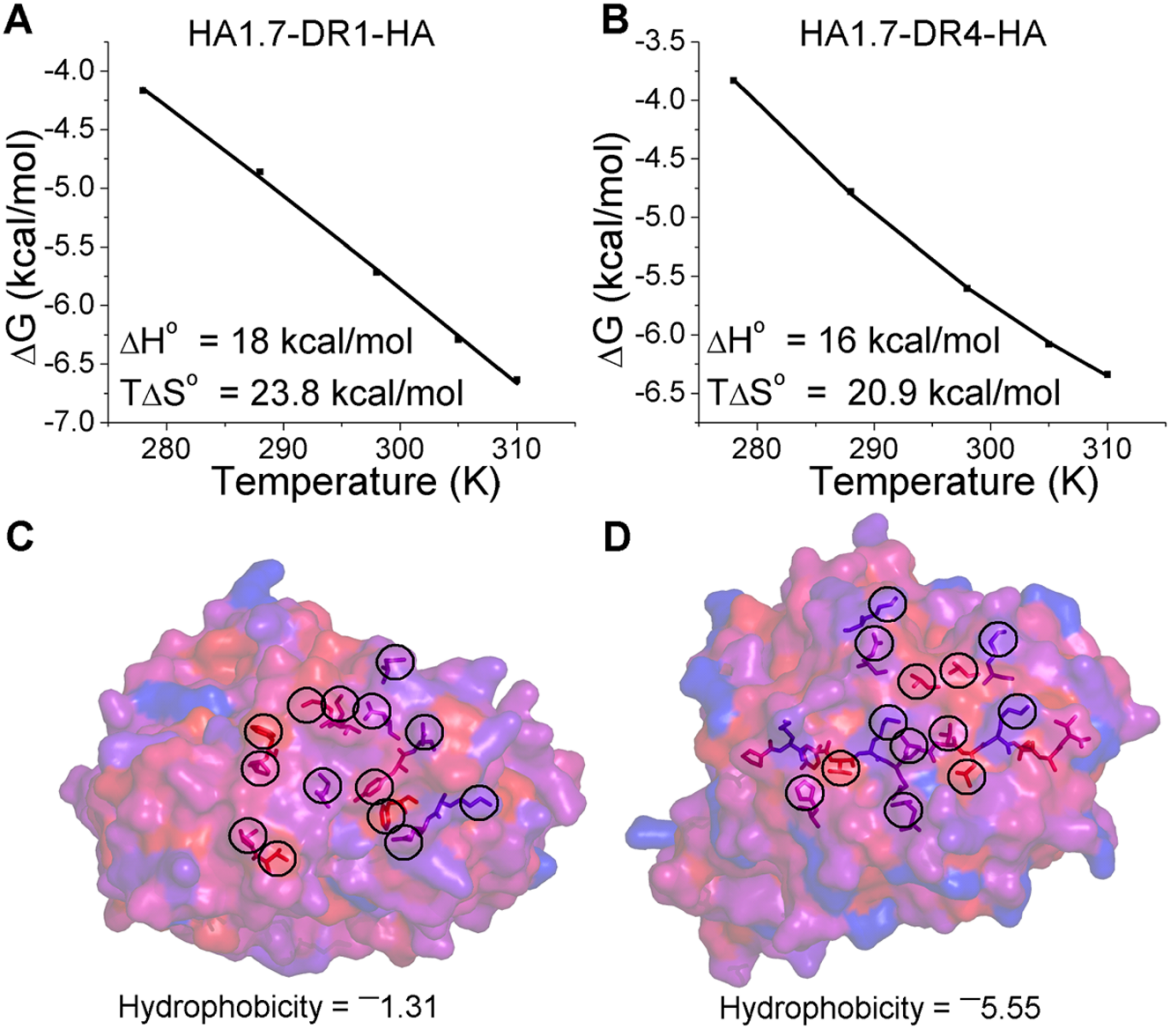
Thermodynamic analyses of HA1.7-DR1-HA and HA1.7-DR4-HA interactions. (A&B) The thermodynamic properties of (A) the HA1.7-DR1-HA and (B) the HA1.7-DR4-HA interactions. Enthalpy (ΔH°) and entropy (TΔS°) at 298 K, are shown in kcal/mol, and were calculated by a non-linear regression of temperature (K) plotted against the free energy (ΔG°). (C&D) Hydrophobic analysis of the residues involved in the binding interface between the HA1.7 TCR and DR1-HA (the hydrophobic characteristics of the DR4-HA surface was virtually identical to DR1-HA). Hydrophobicity was calculated using the normalized consensus hydrophobicity scale as previously described^48^. The TCR and pMHC surfaces are colored according to this scale with blue being the most hydrophobiic and red being the least hydrophobic. In order to calculate the hydrophobicity for each interface, the hydrophobicity scale values for all residues involved in binding were added together. Residues involved in binding are shown as sticks and circled. The full HA peptide is shown as sticks. (C) HA1.7 TCR (PDB: 4GKZ), (D) DR1-HA (cognate pMHC for the HA1.7 TCR) (PDB: 1FYT)^39^.

The vast majority of TCR-pMHC interactions reported are characterized by favorable enthalpy, with entropy playing a more varied role^5^. These studies have provided important molecular information concerning the energetic factors that contribute to T-cell antigen recognition. However, there are currently no published thermodynamic measurements of human MHC-II restricted TCR interactions. Thus, in order to generate the first published report for TCR-pMHC-II interactions, thermodynamic analyses of HA1.7-DR1-HA and HA1.7-DR4-HA were performed. The binding equilibrium (K_D_) was determined at 5 different temperatures for each complex and enthalpy (ΔH^o^), entropy (TΔS^o^) and heat capacity (ΔCp^o^) were calculated by a non-linear regression of temperature (K) plotted against the free energy (ΔG^o^) **(**Figure 4A&B, Supplementary Figure 2**)**. The HA1.7 TCR exhibited extreme thermodynamic parameters compared to previously published TCR-pMHC interactions, with the largest unfavourable enthalpic contribution reported upon binding to both DR1-HA and DR4-HA (ΔH^o^ = 16 and 18 kcal/mol for DR4-HA and DR1-HA, respectively) **(**Figure 4A&B**)**. This large enthalpic penalty indicated a net loss in the number of electrostatic interactions during complex formation. Thus, the greatest reported favourable entropic energy (TΔS^o^ = 20.9 and 23.8 kcal/mol for DR4-HA and DR1-HA, respectively) was required to enable HA1.7 antigen recognition, suggesting a net loss of order during binding (Figure 4A&B). These characteristics are the opposite of what was originally proposed to be the TCR thermodynamic signature (favourable enthalpy and unfavourable entropy) by early thermodynamic TCR-pMHC-I investigations^44^,^45^,^46^. Our observation, that HA1.7 TCR binding to DR1-HA and DR4-HA was entropically driven, also contradicts the prediction by Hennecke *et al* that the binding energy driving the HA1.7-DR1-HA interaction would be derived from the formation of new electrostatic interactions^39^,^40^.

The expulsion of ordered water molecules has been previously implicated as an important mechanism enabling TCR binding to pMHC^47^. Such a loss of ordered water could also partly explain the large favourable entropic energy we observed for HA1.7 binding to DR1-HA and DR4-HA. Ordered water molecules can form a shell around hydrophobic protein patches. Thus, we analyzed the hydrophobic nature of the interface between the HA1.7 TCR and DR1-HA, or DR4-HA using the normalized consensus hydrophobicity scale^48^ (Figure 4C&D). The hydrophobic surface of DR1-HA and DR4-HA were virtually identical, so DR1-HA is described from here on. The HA1.7 TCR was slightly hydrophobic, with residues contributing to the binding interface generating a combined hydrophobicity score of ^−^1.31 (Figure 4C). DR1-HA was more hydrophobic, with a score of ^−^5.55 (Figure 4D). In order to determine whether this level of hydrophobicity was a factor for generating the extreme thermodynamic signature we observed in this system, we compared the hydrophobic interfaces of some other MHC-I restricted TCRs that have been structurally and thermodynamically characterized in previous studies^44^,^49^,^50^,^51^,^52^,^53^. The JM22 TCR bound to HLA A*0201-GILGFVFTL with unfavourable entropy and had a relatively low hydrophobic score (Supplementary Figure 3A&B), whereas the entropically favourable interactions between the LC13 TCR and HLA B*0801-FLRGRAYGL, and the A6 TCR and HLA A*0201-LLFGYPVYV, where substantially more hydrophobic (Supplementary Figure 3C–F). These observations support the notion that a more hydrophobic interface would result in the expulsion of more ordered water molecules upon ligand engagement, generating a larger favourable entropic gain than less hydrophobic interactions. However, the hydrophobic score for the HA1.7-DR1/4-HA interaction was substantially lower than for the other entropically favourable interactions involving the LC13 and A6 TCRs, even though the entropic value for HA1.7 was much more favourable. Thus, hydrophobicity alone could not explain the extreme thermodynamic signature for the HA1.7 TCR. Overall, a combination of a rigid binding mode (limiting entropically unfavourable dissorder to order transition during binding), and the expulsion of some ordered solvent probably explain the extreme thermodynamic parameters that govern HA1.7 TCR ligand engagement and cross reactivity.

## Discussion

Previous structural studies of ligated and unligated MHC-I restricted TCRs have shown that MHC-I antigen recognition can be very dynamic including movements in the TCR CDR loops, peptide, or MHC-I helices^4^,^13^,^14^. However, structural information concerning the molecular mechanism utilized by MHC-II restricted TCRs during antigen engagement is lacking. To this end, we solved the structure of the MHC-II restricted HA1.7 TCR to 2.4Å. It has been shown previously that the DR1 restricted CD4^+^ T-cell clone, HA1.7, can also be activated by HA_306–318 _in the context of DR4^40^ and the structures of both HA1.7-DR1-HA and HA1.7-DR4-HA complexes have been solved^39^,^40^. Thus, this important disease relevant model system was ideal for investigating the structural basis for MHC-II restricted TCR recognition of multiple ligands. Surprisingly, our structural investigation showed that the HA1.7 TCR required only small shifts (up to a maximum of 2.28Å) in TCR CDR loop conformations to form, on average, 50% of the hydrogen bonds and salt bridges upon complex formation, compared to an average of ≥ 5Å for TCR-pMHC-I CDR loop movements^4^. Furthermore, a greater degree of movement was required by the HA1.7 TCR to associate with DR4, which could explain the lower binding affinity between HA1.7 and DR4-HA compared to HA1.7 binding to DR1-HA. These observations may be related to the differences in the antigenic landscape of the pMHC-I and pMHC-II molecules. For instance, the structural database of TCR-pMHC-I and TCR-pMHC-II complexes shows that peptides presented by MHC-I generally assume a central bulged conformation, potentially requiring greater TCR CDR loop movements for engagement compared to TCR binding to the much flatter peptide conformation in the open-ended MHC-II binding groove^39^,^40^,^54^.

The small TCR CDR loop movements required for the HA1.7 T-cell clone to engage with multiple ligands (DR1-HA and DR4-HA) is at odds with previously suggested molecular rules that may govern T-cell degeneracy^8^. In order to examine whether the degenerate nature of the HA1.7 T-cell clone extended beyond the recognition of the DR1-HA and DR4-HA ligands, we altered key TCR contact residues in the HA_306–318 _epitope. Our initial analysis demonstrated that the HA1.7 T-cell clone was able to recognize a number of APLs that contained mutations at key TCR contact residues within the HA_306–318 _epitope. Importantly, the HA1.7 T-cell clone was extremely sensitive to antigen, and could activate against ligands that were too weak to enable tetramer staining using cognate multimerized pMHC-II, and were beyond the detection limits of SPR (data not shown). These findings are in agreement with previous investigations demonstrating that the HA1.7 T-cell clone is highly degenerate and can recognize multiple peptide ligands^21^,^22^. Therefore, our structural analysis indicated that minimal TCR CDR loop movements were sufficient to enable cross-recognition of different ligands by the HA1.7 T-cell clone.

Early thermodynamic analyses of TCR-pMHC-I interactions showed that binding was generally characterized by unfavorable entropy (i.e. transition from a disordered to ordered state) which was counteracted by favorable enthalpy (i.e. exothermic reaction mediated by a net gain in electrostatic interactions)^5^, although more recent studies have shown that the TCR can bind using a range of thermodynamic strategies^5^. Importantly, there are no other reports of TCR-pMHC-II thermodynamics currently in the literature. We performed a thermodynamic analysis of the HA1.7-DR1-HA and HA1.7-DR4-HA interactions in order to further dissect the molecular basis for HA1.7 antigen recognition. This identified an extreme and unusual thermodynamic signature (the largest unfavourable enthalpy and the largest favourable entropy reported). Importantly, the HA1.7 TCR used a virtually identical thermodynamic strategy to bind to both DR1-HA and DR4-HA.

A more in depth analysis of the binding interface demonstrated that the residues involved during HA1.7-DR1/4-HA complex formation were moderately hydrophobic, compared to other entropically favourable TCR-pMHC-I interactions. Previous structural investigations have shown that the LC13 and A6 TCRs undergo conformational changes when engaging different ligands. For example, the CDR loops of the LC13 TCR adopt different conformations in ligated and unligated forms^52^,^55^, and alter their binding mode when engaging different ligands^56^. Similarly, the A6 TCR has recently been shown to undergo conformational melding upon binding to different ligands^7^,^57^. Thus, compared to the rigid binding mode of the HA1.7 TCR, the CDR loops of the LC13 and A6 TCRs may require greater stabilisation during binding. This transition from disorder to order (entropically unfavourable) upon ligand engagement could offset any energetically favourable expulsion of ordered solvent in these systems, compared to the ‘lock and key’ binding of the HA1.7 TCR. Thus, it is likely that a combination of rigid ‘lock and key’ binding, and the expulsion of ordered solvent explain the extreme thermodynamic parameters observed for the HA1.7 TCR. Although the structures of the APLs reported here are not known (and could include large CDR loop movements), this rigid TCR binding mode probably enabled the HA1.7 T-cell clone to cross-react with a range of ligands.

In summary, we have shown that small TCR CDR loop movements and an extreme thermodynamic signature (largest observed unfavourable enthalpy and largest reported favourable entropy) drive the interaction of a MHC-II presented universal haemagglutinin antigen with the HA1.7 TCR. These observations are in contrast to some previously published data for MHC-I restricted TCRs in which large conformational changes in the TCR were deemed important for antigen discrimination and T-cell degeneracy^6^,^8^. Our results show that MHC-II-restricted TCR binding can occur as the result of minimal conformational plasticity and favorable entropy. Although further examples of ligated and unligated MHC-II-restricted TCR structures with different ligands will be required in order to determine whether this is a common theme for TCRs interacting with pMHC-II ligands, we suggest that MHC-II restricted TCRs may employ a distinct binding mode compared to MHC-I restricted TCRs to engage multiple different ligands, perhaps pertaining to the flatter antigenic landscape of pMHC-II molecules compared to pMHC-I.

## Methods

### Generation of expression plasmids

The extracellular constructs of the HA1.7 TCR were designed to incorporate an engineered disulphide link to produce the soluble domains (variable and constant) for both the α and β chains^23^. Sequences for the HA1.7 TCR were cloned and inserted into the pGMT7 expression plasmid allowing protein expression under the control of the T7 RNA polymerase promoter within a Rosetta DE3 *Escherichia coli* system. Plasmid integrity was confirmed by automated DNA sequencing (Central Biotechnology Services, Cardiff University). Expression plasmids encoding extracellular domains of DR1 and DR4 have been described^24^.

### Protein expression, refolding, and purification

Competent Rosetta DE3 *Escherichia coli* cells were induced with 0.5 mM isopropyl β-D-thiogalactoside to produce the HA1.7 TCRα and β chains, the DRα1*0101 chain, the DRβ1*0101 chain and the DRβ1*0401 chain, in the form of inclusion bodies (IBs) as described previously^25^. The DRα1*0101 chain, the DRβ1*0101 chain and the DRβ1*0401 chain were purified into 8 M urea buffer (8 M urea, 20 mM TRIS pH8.1, 0.5 mM EDTA, 30 mM DTT) by ion exchange using a HiTrap^TM^ column (GE Healthcare, UK) to remove bacterial impurities. For a 1 L TCR refold, 30 mg of TCRα chain IBs were incubated for 15 min at 37°C with 10 mM DTT and added to cold refold buffer (50 mM TRIS pH8.1, 2 mM EDTA, 2.5 M urea, 6 mM cysteamine hydrochloride and 4 mM cystamine). After 15 mins, 30 mg of TCRβ chain IBs, also incubated for 15 mins at 37°C with 10 mM DTT, were added. For a 1 L pMHC-II refold, 2 mg of DRα1*0101 chain IBs were mixed with 2 mg of either the DRβ1*0101 chain or the DRβ1*0401 chain IBs and 0.5 mg of peptide for 15 min at 37°C with 10 mM DTT^24^. The HA_306–318 _peptide (PKYVKQNTLKLAT) (generated by Peptide Protein Research Ltd., Southampton, UK) was used in the refold. This mixture was then added dropwise to cold refold buffer (25% glycerol, 20 mM TRIS pH8.1, 1 mM EDTA, 2 mM glutathione reduced, 0.2 mM glutathione oxidized). Refolds were incubated for 72 hr at 4°C. Dialysis was carried out against 10 mM TRIS pH 8.1 until the conductivity of the refolds was <2 mS/cm. The refolds were then filtered, ready for purification steps. Refolded TCR and pMHC-II proteins were then purified initially by ion exchange using a Poros50HQ column and then gel filtrated using a Superdex 200HR column.

### pMHC biotinylation

Biotinylated pMHC-II was prepared either by BirA-mediated enzymatic addition of biotin to bsp-tagged refolded MHC-II-peptide complexes^26^, or by chemical biotinylation using biotin-PEG-maleimide to modify refolded MHC-II-peptide complexes carrying a C-terminal cysteine residue as described previously^27^.

### T-cells

The HA1.7 T-cell clone^28^ provided by Jonathan Lamb, was maintained by biweekly stimulation with HA peptide-pulsed irradiated DR1^+^ antigen presenting cells as described^29^, and rested for 8–10 days before use in activation or tetramer staining assays.

### T-cell proliferation assay

Proliferation was determined by incorporation of [^3^H]-thymidine in peptide-stimulated T-cells using irradiated (4900 rads) DR1^+^ lymphoblastoid cells (LG2) as antigen presenting cells. Briefly, HA1.7 T-cells and LG2 cells were washed 3 times and re-suspended in cRPMI (RPMI 1640 supplemented with 10% FBS, 2 mM L-glutamine, 50 U/ml Penicillin, 50 μg/ml Streptomycin, non-essential amino acids, 1 mM sodium pyruvate and 5×10^−5^ 2-Mercapthoethanol). To initiate the assay, 2×10^4^ T-cells, an equal number of irradiated LG2 cells (2×10^4^), and peptide were added in 200 μl final volume to 96 well round bottom plates. After 24 hours of incubation, 1 μCi/well [^3^H]-thymidine was added, and after additional 24 hours incubation, tritium incorporation was determined by scintillation counting (1450 Microbeta TriLux Scintillation counter, Perkin-Elmer, Shelton, CT).

### IL-2 assay

IL-2 in supernatants was determined by using CTLL-2 cells (TIB-214, ATCC Manassas, VA) as previously described^30^. Briefly, 50 μl of culture medium from stimulated HA1.7 T-cells or controls were transferred to 96 well plates and 1×10^4^ CTLL-2 cells added in medium to bring the final volume to 200 μl. After 24 hours the cells were pulsed with 1 μCi/well [^3^H]-thymidine and incubated for additional 17 hours. Finally, plates were harvested and ^3^H incorporation determined by liquid scintillation counting as above. An IL-2 standard curve was used to relate CTLL-2 proliferation to IL-2 concentration.

### Tetramer staining

Tetramer staining was performed as described previously^27^. Briefly, streptavidin-PE was added step-wise to DR1-HA peptide complexes in 4 aliquots incubating for 2 minutes before addition of next aliquot to obtain a final molar ratio was 4:1. HA1.7 T-cells were incubated with tetramers in PBS+3% BSA for 4 hours at 37°C and for 20 minutes for anti-CD4-APC antibody on ice. Tetramer and antibody binding was determined using a 4 color FACScalibur (BD Biosciences).

### MHC-II-peptide binding analysis

The MHC-II-peptide binding affinity was estimated by competition assay as described^31^. Briefly, 5 nM biotinylated HA peptide and 5 nM DR1, refolded in the absence of peptide^24^, were incubated together with varying concentrations of unlabelled test peptide at 37°C for four days, before measurement of DR1-bioHA by a sandwich microplate immunoassay using the anti-DR1 monoclonal LB3.1 capture and streptavidin-Eu with delayed fluorescence detection. Relative binding affinities are reported as the concentration of test peptide required to inhibit 50% of DR1-bioHA formation, with IC_50_ values determined by fitting to sigmoidal dose-response curve with Hill coefficient = 1. Under these conditions the IC_50 _values approximate the equilibrium dissociation constant K_D_^31^.

### Crystallization, structure determination, and refinement

Refolded HA1.7 TCR was concentrated to 10 mg/ml in 10 mM TRIS pH 8.1 and 10 mM NaCl. Screens were set up in 96 well Intelli-plates (Art Robbins Instruments- ARI) using a crystal phoenix robot (ARI) applying the sitting drop vapour diffusion technique. 0.2 µl HA1.7 TCR and 0.2 µl crystallisation buffer were dispensed into the small reaction well and 60 µl crystallisation buffer dispensed into the large reservoir. Intelli-plates were then sealed and incubated at 18°C in a crystallisation incubator (Molecular dimensions) and analyzed for crystal formation. HA1.7 TCR crystals appeared in 0.1 M Bis-Tris propane pH 8.5, 0.2 M sodium bromide, 20% w/v PEG 3350. Crystals selected for further analysis were cryoprotected with ethylene-glycol to 25% and then flash cooled in liquid nitrogen in Litho loops (Molecular dimensions). Diffraction data was collected at beamline I04.1 at a fixed wavelength of 0.9163 Å at the Diamond light source, Oxford, with a Pilatus 2 M detector. Reflection intensities were estimated with the MOSFLM package^32^ and the data were scaled, reduced and analyzed with SCALA and the CCP4 package^33^. The structure was solved with Molecular Replacement using PHASER^34^. The model sequence was adjusted with COOT^35^ and the model was refined with REFMAC5^36^. Graphical representations were prepared with PYMOL^37^. The reflection data and final model coordinates for the HA1.7 TCR were deposited in the PDB database and assigned accession code: 4GKZ.

### Thermodynamic investigation

Thermodynamic analyses were performed using a BIAcore T100 equipped with a CM5 sensor chip as previously described^38^. 500 response units of biotinylated pMHC-II were immobilized to streptavidin, which was chemically linked to the chip surface. Equilibrium binding analysis was preformed with 10 serial dilutions of the TCR individually injected at teh following temperatures: 5°C, 15°C, 25°C, 32°C, and 37°C. Experiments were performed in triplicate. Representative data are shown. The binding response was determined by subtraction of the response measured on a control flow cell, which contained non-cognate pMHC-II, from the response measured in flow cells containing cognate pMHC-II. Results were analyzed using BIAevaluation 3.1, Microsoft Excel, and Origin 6.1. The equilibrium-binding constant (K_D_) values were calculated using a nonlinear curve fit (y = (P_1_x)/(P_2_ + x)). The thermodynamic parameters were calculated according to the Gibbs-Helmholtz equation (ΔG° = ΔH − TΔS°). The binding free energies, ΔG° (ΔG° = −RTlnK_D_) were plotted against temperature (K) using nonlinear regression to fit the three-parameter equation, (y = dH+dCp*(x-298)-x*dS-x*dCp*ln(x/298)).

## Author Contributions

D.K.C., L.J.S., A.G., A.F., C.J.H, S.V., J.M.C.C., P.J.R., and F.M. performed experiments, analyzed data and critiqued the manuscript. D.K.C., A.G. and L.J.S. conceived and directed the project. D.K.C., L.J.S., A.K.S., A.G., C.J.H, and P.J.R., wrote the manuscript.

## Supplementary Material

Supplementary Information Supplementary Information

## Figures and Tables

**Table 1:** Data collection and refinement statistics (molecular replacement)

Data set statistics	HA1.7 TCR
Space Group	P12_1_1
Unit cell parameters (Å,°)	a = 69.2, b = 49.7, c = 72.6, β 94.3
Radiation Source	DIAMOND I04.1
Wavelength (Å)	0.9763
Resolution (Å)	26.39−2.39
Unique reflections	19445 (1452)
Completeness (%)	100
Multiplicity	3.8 (3.9)
I/Sigma(I)	18.9 (2.3)
Rmerge (%)	0.04 (0.576)
No reflections used	18443
Rcryst (no cutoff) (%)	22.9
Rfree (%)	29.4
Bond lengths (Å)	0.016
Bond Angles (°)	1.761
Mean B value (Å^2^)	22.67
Outliers Ramachandran plot (%)	0
Overall ESU based on Maximum Likelihood (Å)	0.247

*Values in parentheses are for highest-resolution shell.

**Table 2:** CDR loop movement during HA1.7 TCR binding to DR1-HA and DR4-HA

TCR	pMHC-II	CDR loop shift (Å)		
CDR loop		1α	2α	3α
HA1.7	DR1-HA	1.10	1.24	0.83
HA1.7	DR4-HA	0.97	1.25	0.89
CDR loop		1β	2β	3β
HA1.7	DR1-HA	0.97	1.77	1.77
HA1.7	DR4-HA	2.28	1.82	1.92

PyMol software was used to overlap and align the unligated HA1.7 TCR with the two ligated complexes, HA1.7-DR1-HA and HA1.7-DR4-HA, allowing direct visualization of the conformation of the CDR loops before and after MHC-II association. Measurement of individual CDR loop shifts was carried out as previously described^4^.
