# Supplementary material for: Minimal conformational plasticity enables TCR cross-reactivity to different MHC class II heterodimers
Source: Sci Rep. 2012 Sep 4;2:629. doi: 10.1038/srep00629 (PMC3432979; doi:10.1038/srep00629)
Supplement: Supplementary Information [file srep00629-s1.pdf]

Minimal conformational plasticity enables TCR cross-reactivity to different MHC class II heterodimers

Christopher. J. Holland[1], Pierre J. Rizkallah[1], Sabrina Vollers[2], J. Mauricio Calvo-Calle[3], Florian Madura[1], Anna Fuller[1], Andrew K. Sewell[1], Lawrence J. Stern[2,3], Andrew Godkin[1,4#] and David K. Cole[1#*]

[1]Institute of Infection and Immunity, Cardiff University School of Medicine, The Henry Wellcome Building, Cardiff, CF14 4XN, Wales, United Kingdom.

[2]Department of Pathology and [3]Department of Biochemistry & Molecular Pharmacology, University of Massachusetts Medical School, Worcester, MA 01655.

[4]Department of Integrated Medicine, University Hospital of Wales, Cardiff, CF14 4XW, Wales, United Kingdom.

[#]These authors contributed equally

[*]**Corresponding author:** Dr David Cole, Institute of Infection and Immunity, Cardiff University School of Medicine, The Henry Wellcome Building, Cardiff, CF14 4XN, Wales, United Kingdom. E-mail: coledk@cf.ac.uk. Tel: +442920687006.

Keywords: Crystal structure, peptide-major histocompatibility complex class II (pMHC-II), surface plasmon resonance (SPR), thermodynamics, T-cell, T-cell receptor (TCR), influenza, HA1.7

Supplementary Table 1: HA1.7-DR1-HA contacts

CDR loop	TCR	Peptide	MHC	VdW (≤4 Å)	H-bonds (≤3.4 Å)
CDR1α	Val28	Val309		1	
	Pro29		βHis81	1	
CDR3α	Pro96		βGln70	2	
	Phe97		βGln70	2	
	Glu102		αAla61	1	
	Glu102$^{O\varepsilon2}$	Lys310$^{N\zeta}$		1	1 SB
CDR1β	Asp28$^{O/O\delta1}$	Lys315$^{N\zeta}$			2 SB
	Glu30		αAla64	1	
	Glu30$^{O\varepsilon2/O\varepsilon1}$	Lys315$^{N\zeta}$		1	2 SB
CDR2β	Glu56$^{O\varepsilon2}$		αLys39$^{N\zeta}$	1	1 SB
	Glu56$^{O\varepsilon1}$		αGln57$^{N\varepsilon2}$	1	1 HB
	Lys55^{O}		αLys39$^{N\zeta}$		1 HB
	Asp51		αAla64	2	
	Asp51$^{O\delta1}$		αLys67$^{N\zeta}$		1 SB
	Try50		αGln57	1	
	Try50		αAla61	2	
	Phe48		αGln57	1	
CDR3β	Ser96	Lys315		1	
	Thr97^{O}	Asn312$^{N\delta2}$		1	1HB
	Gly98	Thr313		1	
	Gly98	Leu314		1	

H-bonds (HB) = hydrogen bonds, SB = salt bridges, VdW = Van der Waals

Supplementary Table 2: HA1.7-DR4-HA contacts

CDR loop	TCR	Peptide	MHC	VdW (≤4 Å)	H-Bonds (≤3.4 Å)
CDR1α	Pro29		βHis81	4	
	Val28		βHis81	1	
	Val28	Val309		2	
CDR2α	Ala52		βGlu69	1	
CDR3α	Glu102$^{O\varepsilon 2/ O\varepsilon 1}$	Lys310$^{N\zeta}$		1	1 SB
	Phe97		βGln70	5	
	Pro96		βGln70	1	
	Glu94		αGlu55	1	
	Glu94$^{O\varepsilon 2}$	Lys307$^{N\zeta}$			1 SB
CDR1β	Glu30		αAla64	1	
	Glu30$^{O\varepsilon 1/O\varepsilon 2}$	Lys315$^{N\zeta}$		1	2 SB
	Asp28$^{O/O\delta 1}$	Lys315$^{N\zeta}$			2 SB
CDR2β	Glu56$^{O\varepsilon 2}$		αLys39$^{N\zeta}$		1 SB
	Lys55^{O}		αLys39$^{N\zeta}$		1 HB
	Met54		αLeu60	1	
	Asp51		αAla64	2	
	Asp51$^{O\delta 1/ O\delta 2}$		αLys67$^{N\zeta}$		1
	Try50		αAla61	3	
CDR3β	Gly98	Leu314		1	
	Pro100		βGln64	1	
	Pro100		βLeu67	2	

H-bonds (HB) = hydrogen bonds, SB = salt bridges, VdW = Van der Waals

Supplementary Figure 1. HA1.7 T-cell recognition of DR1-HA and altered peptide ligands

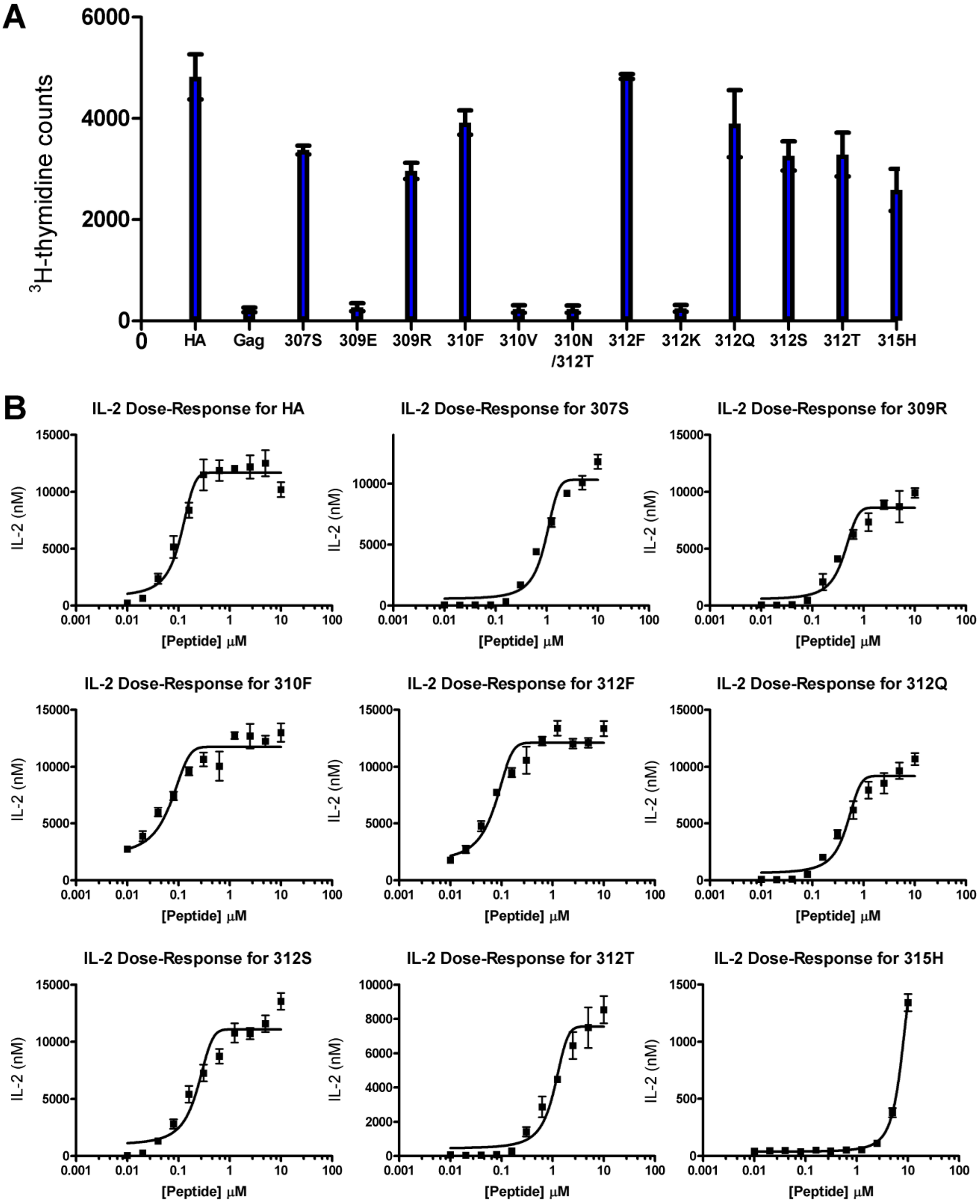

Supplementary Figure 1. **(A)** HA1.7 T-cell proliferation assay using DR1 presenter cells pulsed with; wild-type HA peptide (positive control), a non-cognate Gag peptide (negative control), or an altered peptide ligand. **(B)** HA1.7 T-cell IL-2 production using DR1 presenter cells pulsed with; wild-type HA peptide (positive control), or an altered peptide ligand. APLs 309E, 310V, 310N/312T and 312K did not induce IL-2 release (data not shown).

Supplementary Figure 2. Thermodynamic analysis of HA1.7 TCR binding to DR1-HA and HLA-DR4-HA

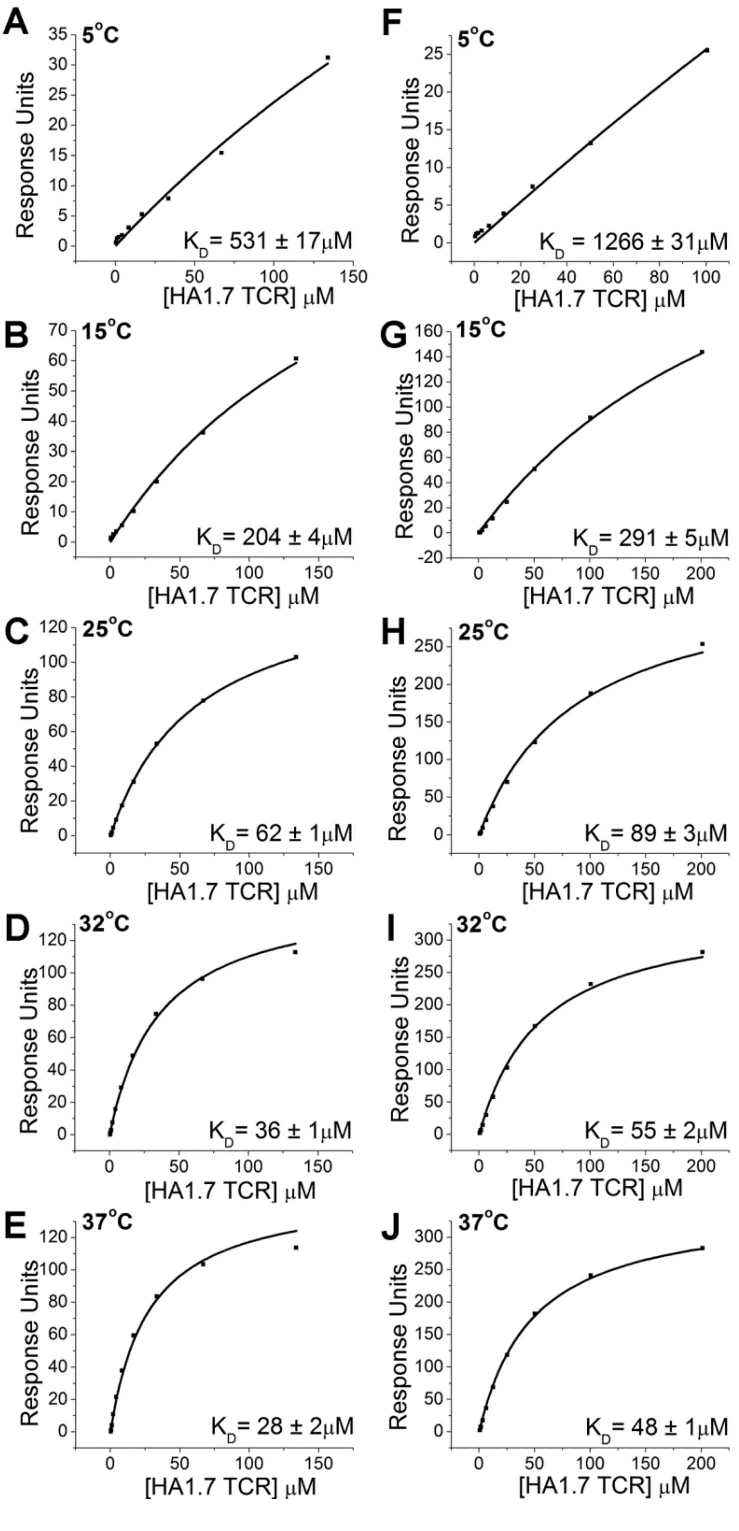

Supplementary Figure 2. **(A-J)** Equilibrium-binding analysis of HA1.7-DR1-HA (left column) and HA1.7-DR4-HA (right column) at **(A&F)** 5°C, **(B&G)** 15°C, **(C&H)** 25°C, **(D&I)** 32°C and **(E&J)** 37°C for thermodynamic analysis. These data were performed in triplicate. Representative data are shown. Ten serial dilutions were conducted for each equilibrium experiment. The equilibrium binding constant (K_D) values are plotted using a nonlinear curve fit ($y = (P_1x)/(P_2 + x)$).

Supplementary Figure 3. Hydrophobic analysis of TCR and pMHC binding interfaces

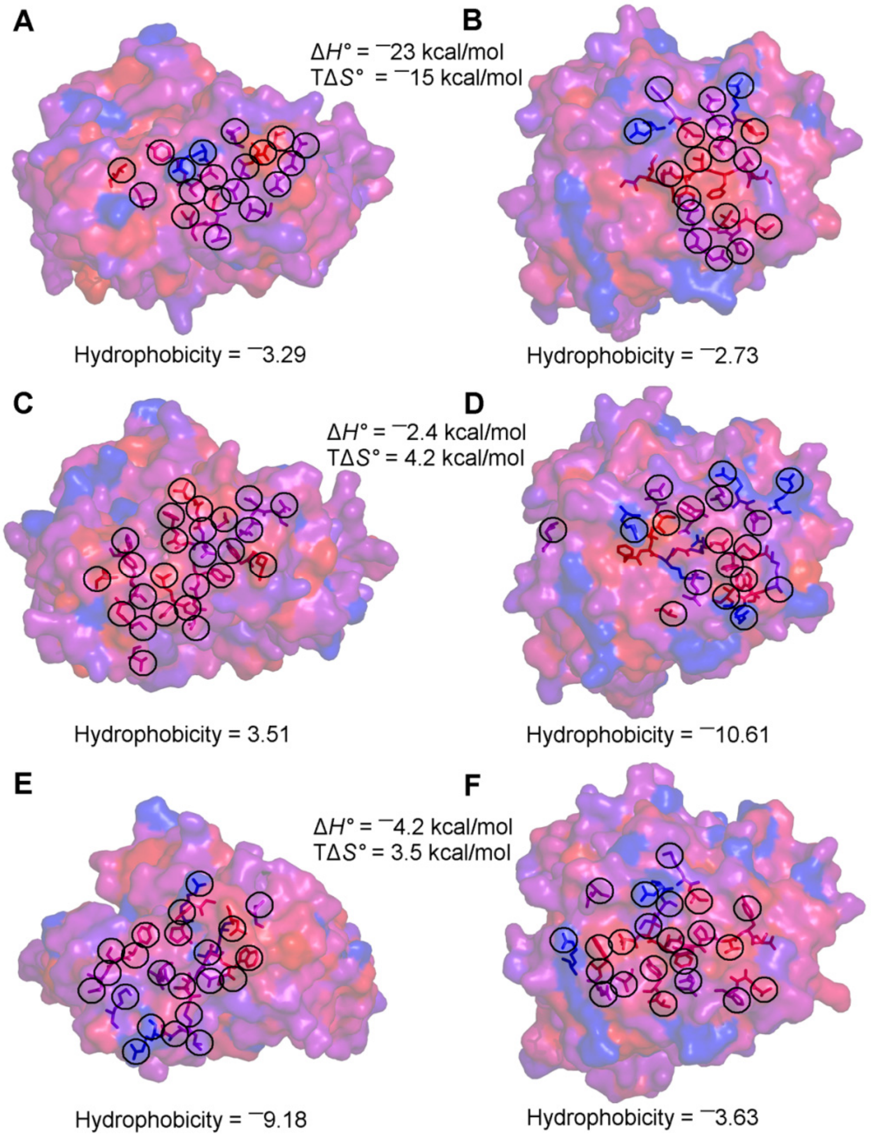

Supplementary Figure 3. Hydrophobic analysis of the residues involved in the binding interface between different TCR-pMHC complexes. Hydrophobicity was calculated using the normalized consensus hydrophobicity scale as previously described[1]. The TCR and pMHC surfaces are colored according to this scale with blue being the most hydrophobiic and red being the least hydrophobic. In order to calculate the hydrophobicity for each interface, the hydrophobicity scale values for all residues involved in binding were added together. In all cases, residues involved in binding are shown as sticks and circled. Full peptides are shown as sticks in all cases. **(A)** JM22 TCR, **(B)** HLA A*0201-GILGFVFTL (cognate pMHC for the JM22 TCR) (PDB: 1OGA). **(C)** LC13 TCR, **(D)** HLA B*0801-FLRGRAYGL (cognate pMHC for the LC13 TCR) (PBD: 1MI5). **(E)** A6 TCR, **(F)** HLA A*0201-LLFGYPVYV (cognate pMHC for the A6 TCR) (PDB: 1AO7)[2]. Thermodynamic parameters shown were calculated from previously published studies[3-5].

References:

1. Eisenberg, D., Schwarz, E., Komaromy, M. & Wall, R. Analysis of membrane and surface protein sequences with the hydrophobic moment plot. *J Mol Biol* **179**, 125-142 (1984).
2. Garboczi, D.N. *et al.* Structure of the complex between human T-cell receptor, viral peptide and HLA-A2. *Nature* **384**, 134-141 (1996).
3. Davis-Harrison, R.L., Armstrong, K.M. & Baker, B.M. Two different T cell receptors use different thermodynamic strategies to recognize the same peptide/MHC ligand. *J Mol Biol* **346**, 533-550 (2005).
4. Ely, L.K. *et al.* Disparate thermodynamics governing T cell receptor-MHC-I interactions implicate extrinsic factors in guiding MHC restriction. *Proc Natl Acad Sci U S A* **103**, 6641-6646 (2006).
5. Willcox, B.E. *et al.* TCR binding to peptide-MHC stabilizes a flexible recognition interface. *Immunity* **10**, 357-365 (1999).
